# Long-term course and factors influencing work ability and return to work in post-COVID patients 12 months after inpatient rehabilitation

**DOI:** 10.1186/s12995-024-00443-4

**Published:** 2024-11-01

**Authors:** Katrin Müller, Iris Poppele, Marcel Ottiger, Alois Wastlhuber, Rainer-Christian Weber, Michael Stegbauer, Torsten Schlesinger

**Affiliations:** 1 Institute of Human Movement Science and Health, Faculty of Behavioral and Social Sciences, Chemnitz University of Technology, 09107 Chemnitz, Germany; 2 BG Hospital for Occupational Disease Bad Reichenhall, 83435 Bad Reichenhall, Germany

**Keywords:** post-COVID, Work ability, Physical capacity, Neuropsychological health, Fatigue, Rehabilitation

## Abstract

**Background:**

Rehabilitation plays a crucial role in restoring work ability and facilitating the reintegration of post-COVID patients into the workforce. The impact of rehabilitation on work ability and return to work (RTW) of post-COVID patients remains poorly understood. This study was conducted to assess the work ability and RTW of post-COVID patients before rehabilitation and 12 months after rehabilitation and to identify physical and neuropsychological health factors influencing RTW 12 months after rehabilitation.

**Methods:**

This longitudinal observational study included 114 post-COVID patients with work-related SARS-CoV-2 infection who underwent inpatient post-COVID rehabilitation with indicative focus on pulmonology and/or psychotraumatology (interval between date of SARS-CoV-2 infection and start of rehabilitation: M = 412.90 days). Employment status, work ability, and the subjective prognosis of employment (SPE) scale were assessed before rehabilitation (T1) and 12 months after rehabilitation (T4). The predictors analysed at T4 were functional exercise capacity, physical activity, subjective physical and mental health status, fatigue, depression, and cognitive function. Longitudinal analyses were performed via the Wilcoxon signed-rank test. Logistic and linear regression analyses identified predictors of work ability and return to work (RTW), whereas mediation analyses examined the relationships between these predictors and work ability.

**Results:**

At T4, the median of WAI total score indicated poor work ability, which significantly worsened over time (*p* < 0.001; *r* = 0.484). The SPE scale significantly increased from T1 to T4 (*p* = 0.022, *r* = -0.216). A total of 48.6% of patients had returned to work 12 months after rehabilitation. Fatigue was identified as the main predictor of reduced work ability and RTW, with each unit increase in fatigue severity decreasing the odds of RTW by 3.1%. In addition, physical capacity and subjective health status were significant predictors of perceived work ability.

**Conclusions:**

The findings highlight the significant challenges that post-COVID patients face in regaining work ability and achieving successful RTW 12 months after rehabilitation. Fatigue appears to be an important predictor of work ability and RTW. To optimize recovery and enhance both biopsychosocial health and work ability, it is crucial to develop and implement personalised interventions that address fatigue, improve physical capacity, and support mental health.

**Trial registration:**

This study is registered in the German Clinical Trials Register under DRKS00022928.

**Supplementary Information:**

The online version contains supplementary material available at 10.1186/s12995-024-00443-4.

## Background

The global COVID-19 pandemic has significantly affected workplaces, increasing the risk of SARS-CoV-2 transmission due to frequent interpersonal contact [1, 2]. A study across various occupational sectors, including healthcare and business management, has revealed elevated infection rates among essential workers [3]. In particular, healthcare workers face a greater risk of contracting COVID-19 compared to the general population [4]. In Germany, 359.763 cases of COVID-19 were recognized as occupational diseases and 27.069 cases were recognized as work-related accidents until June 2024, according to the German Social Insurance Code (§ 9 SGB VII) [5] and the Ordinance on Occupational Diseases (“Berufskrankheiten-Verordnung – BKV”, Appendix 1) [6]. In the aftermath of acute SARS-CoV-2 infection, approximately 3–10% of infected individuals can experience persistent post-COVID symptoms [7–9]. Fernandez-de-Las-Peñas et al. [10] reported, that post-COVID-19 symptoms can persist even two years post infection. Research has revealed three main post-COVID-19 symptom clusters: persistent fatigue, cognitive problems, and ongoing respiratory problems [11, 12]. Persistent post-COVID symptoms not only affect physical and mental well-being but also significantly impact an individual’s ability to work or return to work (RTW) after infection [13–16].

The concept of work ability encompasses a range of factors, including physical and mental health, functional capacities, competencies, values, attitudes, and motivation, as well as the demands, arrangements, and management of work [17]. Achieving a balance between these human and work-related factors is crucial for maintaining optimal work ability, both in general and specifically in the context of recovering from COVID-19. Research has highlighted the challenges individuals face in maintaining or regaining their work ability after experiencing COVID-19. Green et al. [18] underscore the challenges faced by individuals with post-COVID, with a substantial proportion experiencing work-related difficulties. Among those participants (*n* = 214), 18% were working, 40% were working with difficulties, and 35% had stopped working due to symptoms like fatigue, which significantly impacted their work ability and quality of life. Similarly, Harvey-Dunstan et al. [19] revealed an association between fatigue and lower likelihood of returning to previous work hours. Moreover, Kerksieck et al. [20] demonstrated that post-COVID symptoms reduced work ability, particularly for physical and mental demands, with older age and psychiatric history worsening the impact. Braig et al. [21] reported that 13.1% of over 9.000 employers noted low Work Ability Index (WAI) score, with factors like medical treatment and intensive care during infection contributing to reduced work ability. Additionally, structural changes within the workplace, including modifications to physical workspaces, adjustments to tasks and workload, and flexible work arrangements, have been identified as facilitators of post-COVID work ability [22]. However, despite such adjustments, the WAI among post-COVID individuals remains suboptimal, as evidenced by the study’s findings of a mean WAI of 24.9, indicating poor work ability. These results were confirmed by Müller et al. [23]. Furthermore, the economic consequences of post-COVID work disability are substantial. Studies have estimated significant losses of productivity in companies and financial burdens on healthcare and social welfare systems due to COVID-19-related work absences and disability [24–27].

RTW after SARS-CoV-2 infection presents another complex challenge. Individuals may face various challenges, including physical, psychological, and cognitive barriers, and workplace accommodations. Aben et al. [28] conducted a study comprising two groups of employees: those on sick leave due to COVID-19 (*n* = 30.396), and those on sick leave due to flu-like symptoms not attributable to COVID-19 (*n* = 15.862). While the RTW rate three months after the onset of flu-like symptoms was 100%, it was 92.8% after the onset of COVID-19. Predictors of delayed RTW included older age, female sex, belonging to a risk group (e.g., chronic diseases), specific symptoms such as shortness of breath and fatigue, prior sick leave, and inpatient care [29, 30]. However, temporal trends suggest improvements in RTW rates as different virus variants become dominant [28]. Moreover, the availability of supportive resources, including healthcare services, rehabilitation programs, and workplace accommodations, plays a crucial role in facilitating successful RTW outcomes [31]. However, disparities in access to these resources and varying levels of employer support can exacerbate challenges for some individuals, delaying their RTW. Unoki et al. [32] reported that RTW rates varied over time following COVID-19 infection, with approximately one-third of COVID-19 patients did not return to work 12 months after receiving intensive care. Furthermore, Rahmati et al. [33] reported that 14.1% of over 1 million participants were unable to return to work even 2 years after SARS-CoV-2 infection, indicating that delayed RTW can persist in the long-term. Understanding the interplay between post-COVID symptoms, work ability, and RTW dynamics is essential for developing comprehensive strategies to support individuals in their recovery journey, promote workplace inclusion and productivity, and mitigate the broader economic impacts of post-COVID. Rehabilitation can play a pivotal role in restoring work ability following SARS-CoV-2 infection [34]. Despite limited research on this topic, our study aims to address the impact of rehabilitation on work ability and RTW in post-COVID patients, evaluating work ability and RTW before rehabilitation and 12 months after rehabilitation. Additionally, the study seeks to analyse group differences in work ability and RTW regarding the participation in aftercare interventions until 12 months after rehabilitation to identify potential associations. Furthermore, we aimed to identify physical and neuropsychological health factors that influence work ability and RTW 12 months post-rehabilitation. By analysing how these factors interact with work ability and RTW, we aim to provide a better understanding of the individual needs of post-COVID patients and support efforts to mitigate the long-term effects of post-COVID on work ability. This comprehensive approach will not only expand the existing knowledge on post-COVID recovery but also aid in the development of individualized rehabilitation programs that address the specific challenges faced by this population.

## Methods

This study (German Clinical Trials Register: DRKS00022928) was conducted at the Chemnitz University of Technology in Germany in collaboration with the BG Hospital Bad Reichenhall. The study was approved by the Ethics Committee of the Bavarian State Medical Association (number 21092) and the Ethics Committee of the Chemnitz University of Technology (TU Chemnitz, Chemnitz, Germany), Faculty of Behavioral and Social Sciences (number V-427-17-KM-COVID-19-18022021). The detailed study protocol was published previously [35]. Only information pertinent to the current research question is presented here.

### Study design and participants

This prospective, longitudinal observational study employed four different measurement time points: at the beginning (T1) and end (T2) of the inpatient rehabilitation period, as well as 6 months (T3) and 12 months (T4) after the beginning of rehabilitation. Only patients in the post-acute phase of COVID-19 (> 3 months after SARS-CoV-2 infection), recognized as an occupational disease or work-related accident could registered for rehabilitation at the German BG Hospital Bad Reichenhall by their respective professional association or accident insurance providers. After that, patients were involved in the study by a study nurse. Following eligibility screening, patients provided written informed consent. All participants underwent a multidisciplinary post-COVID rehabilitation program at BG Hospital, with an average duration of 28.80 days (standard deviation (SD): 5.23 days). Detailed information on the comprehensive multimodal and interdisciplinary inpatient rehabilitation program can be found at Müller et al. [35] and Müller et al. [36]. Additionally, the results regarding physical and neuropsychological health and work ability at measurement points T1 and T2 are published in Müller et al. [23].

The study initially included 127 patients (97 females and 30 males) at T1. Until T4 12 participants were classified as dropouts due to the following reasons: discontinuation of rehabilitation (*n* = 1), lack of interest in continuing the study (*n* = 6), health and time constraints as reasons for withdrawing from that specific assessment (*n* = 2), without providing reasons (*n* = 2), professional reasons (*n* = 1). After accounting for these dropouts and one missing questionnaire related on the main outcome of work ability, paired sample analyses were conducted for 114 participants. Due to missing values, the number of cases for each variable ranged from 90 to 114.

The mean (M) age of the 114 included patients (female: *n* = 86) was 50.62 years (SD: 10.85). The Body-Mass-Index (BMI) was M = 31.34 kg/m^2^ (SD: 6.27). At T1, 85.1% of patients were classified as overweight (BMI > 25 kg/m^2^). Specifically, the distribution of BMI classifications was as follows: 17 individuals (15.0%) were classified as normal weight, 38 (33.3%) as overweight, 32 (28.1%) as class I obesity, 18 (15.8%) as class II obesity, and 9 (7.9%) as class III obesity. During the acute stage of SARS-CoV-2 infection, 82 patients experienced a mild to moderate disease, 27 patients experienced a severe disease (oxygen saturation < 90% on room air, signs of severe respiratory distress or signs of pneumonia), and 5 patients experienced a critical course of disease (life sustaining treatment is required, acute respiratory distress syndrome, sepsis or septic shock). There were no data of the use of Paxlovid^®^ during acute illness. The time interval between date of SARS-CoV-2 infection and start of rehabilitation was 412.90 days (SD: 143.61 days) on average. The patients received after inpatient rehabilitation until 12 months after rehabilitation discharge (T4) the following individual medically prescribed inpatient and outpatient post-COVID-19 treatments: 96 required further medical treatment (e.g. general medicine, neurology, pneumology, psychiatry), 54 were prescribed outpatient psychological therapy, 91 made use of ambulatory active physiotherapy, 28 repeated an in- or outpatient rehabilitation, and 18 were exercising in an outpatient group. Among the included patients, 80 were employed in the healthcare sector, whereas 34 were classified as nonhealthcare workers, regardless of their ability to work. According to the socioeconomic status (SES) scale, 37 patients had a medium socioeconomic status, and 76 patients had a high socioeconomic status. In terms of preexisting conditions, a significant number of patients reported preexisting conditions: 72 (63.2%) had metabolic diseases, 54 (47.4%) had cardiovascular diseases, 49 (43.0%) experienced respiratory diseases, 74 (65.0%) suffered from musculoskeletal diseases, 21 (18.4%) had psychological conditions, and 38 (33.3%) reported neuro-sensory diseases. A detailed description of the included study population is provided in Table 1.

**Table 1:** Sociodemographic data at T1 (*N* = 114)

	*N* (%)	M	SD	95%CI	NA^1^
Sex					
Male	28 (25)				
Female	86 (75)				
Age [years]		50.54	10.85	48.72–52.43	
BMI [kg/m²]		31.34	6.27	30.35–32.68	
Normal (18.5–24.9 kg/m²)	17 (15)				
Overweight (25.0–29.9 kg/m²)	38 (33)				
Obesity class I (30.0–34.9 kg/m²)	32 (28)				
Obesity class II (35.0–39.9 kg/m²)	18 (16)				
Obesity class III (> 40 /m²)	9 (8)				
Smoking status					
Currently (every day)	5 (4)				
Currently (occasional)	4 (4)				
Former	42 (37)				
Never	63 (55)				
COVID-19 severity					
Mild/moderate	82 (72)				
Severe	27 (24)				
Critical	5 (4)				
Pneumonia due to COVID-19	32 (28)				
Treatment in intensive care unit	7 (6)				
Interval COVID-19 to Rehabilitation [days]		412.90	143.61	382.69–434.17	
Rehabilitation duration [days]		28.80	5.23	27.84–29.73	
PCS score					2
No/mild PCS	1 (1)				
Moderate PCS	4 (4)				
Severe/relevant PCS	107 (95)				
Occupation					
Healthcare worker	80 (70)				
Nonhealthcare worker	34 (30)				
RTW at T4					
Yes	54 (47)				
No	57 (50)				
In reintegration process	6 (5)				
Disability pension	8 (7)				
Old-age pension	3 (3)				
Socioeconomic status					1
Medium	37 (33)				
High	76 (67)				
Preexisting diseases					
Metabolic disease	72 (63)				
Cardiovascular disease	54 (47)				
Respiratory disease	49 (43)				
Muscle sceletal disease	74 (65)				
Psychological disease	21 (18)				
Neuro-sensory disease	38 (33)				
Aftercare interventions					
Further medical treatment	96 (86)				2
Repeated rehabilitation	28 (25)				3
Exercising in an outpatient group	18 (17)				9
Ambulatory active physiotherapy	91 (82)				3
Outpatient psychological therapy	54 (50)				5

M - Mean, SD - Standard Deviation, ^1^NA=not available/no answer BMI – Body-Mass-Index, PCS - Post-Covid Syndrome, RTW - Return to work

### Measurements

#### Sociodemographic variables, and anamnesis

Several sociodemographic variables, such as age, sex (self-reported), SES, education, employment, and occupation, were collected via questionnaires based on the German Health Interview and Examination Survey for Adults (DEGS) [37, 38]. The participants assessed their social status using the subjective social ladder from the German version of the MacArthur Scale [39, 40]. The presence of post-COVID symptoms was documented using a questionnaire developed specifically for this study in the basis of valid guidelines [7] and differentiated into symptom clusters based on Bahmer et al. [41]. The Post-Covid Syndrome (PCS) score was also calculated in accordance with the methodology proposed by Bahmer et al. [41]. The PCS score is used to distinguish between no/mild PCS (≤ 10.75), moderate PCS (> 10.75 to ≤ 26.25), and severe/relevant PCS (> 26.25). Additionally, a semi-standardized interview conducted by a physician during medical anamnesis evaluated preexisting medical conditions and the BMI. In addition, at time point T4, patients were asked by questionnaire to indicate post-COVID-19 treatments after inpatient rehabilitation (e.g., follow-up treatment by the general practitioner, ambulatory active physiotherapy (e.g., breathing therapy), or exercising in an outpatient group).

#### Employment and work ability

The perceived ability to work was evaluated via the WAI [42], a widely recognized, valid, and reliable tool that assesses workers’ health status, job demands, and available resources. The WAI consists of seven subscales, each containing one or more questions: (Dimension 1) current work ability compared to the lifetime best; (Dimension 2) ability to work in relation to job requirements; (Dimension 3) number of physician-diagnosed diseases; (Dimension 4) estimated loss of work ability due to illness; (Dimension 5) absence from work in the past year; (Dimension 6) the worker’s own prognosis of future work ability; (Dimension 7) mental resources. The overall index is calculated by summing the points from each item, resulting in a score that classifies work ability as poor (7–27 points), moderate (28–36 points), good (37–43 points), or excellent (44–49 points) [43].

The German version of the subjective prognosis of employment (SPE) scale was used to predict the subjective prognosis of employment and consists of three items. Each item is rated on a binary scale, with 0 or 1 points allocated, and focuses on the following aspects: [1] expectation of employment until retirement; [2] permanent threat to work ability; and [3] consideration of applying for disability pension. The SPE scale has a range from 0 to 3 and a higher score is indicating higher endangerment of employment [44].

During the anamnesis at the rehabilitation clinic, the medical staff documented whether the patient was declared as able or unable to work (RTW: Yes/No) at T1 and T4 by an external assessment for work ability by the treating physician at the place of residence.

#### Physical health, fatigue, and neuropsychological health

The six-minute walking test assessed the functional exercise capacity by measuring the distance the patients could walk in six minutes (6MWD) [45].

To assess patients’ physical activity 12 months after rehabilitation discharge, they were asked to wear an accelerometer (Actigraph GT9X Link ©) on their right waist for 7 days 24 h/day. The recorded physical activity (PA) was categorised into four activity intensities: inactive (< 1.5 metabolic equivalent of task (MET)), light (≥ 1.5 MET), moderate (≥ 3 MET) and vigorous (≥ 6 MET) [46]. A detailed description of the analysis of the accelerometric data is published elsewhere [47]. For this analysis, the time spent in physical inactivity per day (PIA) and the time in moderate to vigorous physical activity (MVPA) were used.

The subjective perceived status of physical and mental health (SPSH) was reported by participants on a self-generated questionnaire (Supplementary Material 2) with 20 items on a scale of 0–10 (0 = very bad, 10 = very well). The total score is given as the mean value.

The Fatigue Impact Scale (FIS) includes 40 items assigned to the three subscales (Dimension 1: cognitive functioning; Dimension 2: physical functioning; Dimension 3: psychosocial functioning). Respondents answer questions on a five-point Likert scale, resulting in a sum score ranging from 0 to 160. Higher scores indicate more severe functional impairments due to fatigue [48].

The subscale for depression (HADS-D_Depression_) of the German version of the Hospital Anxiety and Depression Scale was used to assess the presence of depressive symptoms. The subscale consists of seven items. A sum score was generated (range 0–21) on the basis of responses to 7 items on a Likert scale [49, 50].

The Digit Symbol Substitution Test (DSST; [51]) was applied to assess cognitive functions, including motor speed, attention and visuoperceptual functions. Participants matched symbols to digits (1 to 9) on a sheet for 90 s, with the number of correct matches recorded (DSST1). After that, the test participants wrote down the correct symbols for each digit from memory on a separate page, and the number of correct responses (DSST2) was noted [51].

### Statistical analyses

The data were analysed using SPSS software (version 29, SPSS Inc., Armonk, NY, USA). Given the nonnormal distribution of most parameters, the Wilcoxon signed-rank test was used to compare variables (WAI and SPE) across T1 and T4. Group differences concerning aftercare interventions were analysed using the Mann–Whitney U test. Only significant results regarding group differences at T4 (Supplementary Material 1: Table A1, A2, A3 and A4) are presented in the text. Missing data were noted and are clearly presented in the tables, and *p*-values < 0.05 were considered statistically significant. Effect sizes were reported as r, with an effect size of 0.1 representing a ‘small’ effect, 0.3 representing a ‘medium’ effect, and 0.5 representing a ‘large’ effect, following the guidelines of Fritz et al. (2012). Furthermore, to identify any potential influencing factors on RTW 12 months after rehabilitation (T4), a logistic regression was conducted, with the outcome measure of RTW (yes/no) at T4. First, separate bivariate logistic regressions were conducted between the outcome variable and potential predictors (age, sex, BMI, COVID-19 severity, professional group, PCS score, physical capacity, severity of depressive symptoms, fatigue severity, SPSH, cognitive function (processing speed and memory performance), PIA per day, MVPA per day) measured at T4. The descriptive values of the potential predictors at T4 are reported in Supplementary Material 1, Table A5. Next, the significant predictors were included in a stepwise logistic regression (forward selection and likelihood ratio test). The Nagelkerke R^2^ is considered the effect size of the explanation of variance. The interpretation is analogous to that of Backhaus et al. [52] with Nagelkerke R^2^ > 0.2 as a small effect, R^2^ > 0.4 as a medium effect and R^2^ > 0.5 as a large effect. To identify the factors influencing perceived ability to work, multiple linear regression analyses were conducted (outcome: WAI total at T4). In the first step, bivariate Spearman correlations were performed. In a subsequent step, the parameters with significant correlations were included as potential predictors in the stepwise multiple linear regression analyses after checking the test conditions (homoscedasticity, no multicollinearity (variance inflation factor (VIF) < 10)). The adjusted R^2^ was used as the coefficient of determination and was interpreted as follows: low goodness-of-fit (R^2^_adj_ = 0.02), moderate goodness-of-fit (R^2^_adj_ = 0.13) and high goodness-of-fit (R^2^_adj_ = 0.26) according to Cohen [53]. The applied feature selection method was used to identify as few predictors as possible that still allow for an accurate prediction of the criteria (WAI total and RTW at T4) [54]. Finally, to deepen the understanding of the underlying relationships between the outcome variable (WAI total at T4) and the detected predictors, mediation analyses were conducted. A mediation effect was considered significant if the corresponding bootstrapped (5000 bootstrap samples) 95% confidence interval (CI) of the indirect effect excluded zero. For the mediation analyses the PROCESS macro for SPSS (version 4.2) was used.

## Results

### Work ability and subjective prognosis of employment over time

At T4, 54 (47%) post-COVID patients had returned to work, whereas 57 (50%) did not. Among the patients who were unable to work, 6 (5%) were in the process of reintegration, and an additional 8 (7%) patients were receiving disability pension and 3 (3%) patients old-age pension. The results of the perceived ability to work (WAI) and the subjective prognosis of employment (SPE) scale at T1 and T4 are presented in Table 2. The results highlight significant changes in various dimensions of work ability and employment prognosis over time. At T1, the patients reached Mdn = 2 (IQR 2.00–3.00) points in WAI Dimension 3. WAI Dimension 3 decreased significantly at T4 to Mdn = 2.00 (IQR 2.00–2.00) (*p* < 0.001, *r* = -0.449). WAI Dimension 4 exhibited a significant decrease from Mdn = 3.50 (IQR 2.00–4.75) points at T1 to Mdn = 2.00 (IQR 1.00–4.00) points at T4 (*p* < 0.001, *r *= -0.466). WAI Dimension 6 significantly decreased over time (T1: Mdn = 4.00, IQR 4.00–4.00 points; T4: Mdn = 4.00, IQR 1.00–4.00 points; *p* = 0.004, *r* = -0.274). The overall WAI total score (sum of Dimensions 1–7) significantly decreased from Mdn = 24.50 (IQR 20.00–28.00) points at T1 to Mdn = 21.00 (IQR 17.00–27.00) points at T4 (*p* < 0.001) with a medium effect size (*r* = 0.484). No other significant results were observed (see Table 2). Finally, the SPE scale significantly increased from Mdn = 2.00 (IQR 1.00–3.00) points at T1 to Mdn = 2.00 (IQR 1.00–3.00) points at T4 (*p* = 0.022, *r *= -0.216).

**Table 2:** Differences in work ability and subjective prognosis of employment between T1 and T4

	*N*	T1			T4			z	*p*	*r*
		Min	Max	Mdn (IQR)	Min	Max	Mdn (IQR)			
WAI Dimension 1 (0–10)	113	0.00	9.00	3.00 (1.00–5.00)	0.00	10.00	3.00 (0.00–6.00)	-0.038	0.969	-0.004
WAI Dimension 2 (2–10)	113	3.00	10.00	7.00 (6.00–9.00)	2.00	10.00	7.00 (6.00–8.00)	-1.597	0.110	-0.149
WAI Dimension 3 (1–7)	114	2.00	7.00	2.00 (2.00–3.00)	2.00	7.00	2.00 (2.00–2.00)	-4.789	< 0.001	-0.449
WAI Dimension 4 (1–6)	112	1.00	6.00	3.50 (2.00–4.75)	1.00	6.00	2.00 (1.00–4.00)	-4.928	< 0.001	-0.466
WAI Dimension 5 (1–5)	113	1.00	5.00	2.00 (1.00–2.00)	1.00	5.00	1.00 (1.00–2.00)	-1.004	0.315	-0.094
WAI Dimension 6 (1–7)	111	1.00	7.00	4.00 (4.00–4.00)	1.00	7.00	4.00 (1.00–4.00)	-2.887	0.004	-0.274
WAI Dimension 7 (1–4)	113	1.00	4.00	2.00 (2.00–3.00)	1.00	4.00	2.00 (2.00–3.00)	0.000	1.000	0.000
WAI Total (7–49)	111	14.00	35.00	24.50 (21.00–28.00)	14.00	35.00	21.00 (17.00–27.00)	-5.098	< 0.001	-0.484
SPE scale (0–3)	112	0.00	3.00	2.00 (1.00–3.00)	0.00	3.00	2.00 (1.00–3.00)	-2.285	0.022	-0.216

WAI – Work ability index, SPE – subjective prognosis of employment, Mdn – Median, IQR – Interquartile range

Group differences over time revealed a significant difference in WAI Dimension 5 regarding psychological treatment (yes vs. no) (*p* = 0.027, *r* = 0.213). Post-COVID patients who were undergoing psychotherapeutic treatment showed less improvement in absence from work in the past year (ΔMdn = 0.00, IQR -1.00–0.00) than post-COVID patients without psychotherapeutic treatment (ΔMdn = 0.00, IQR -1.00–1.00). The Tables A1, A2, A3 and A4 in the Supplementary Material 1 provide a comprehensive overview of the results and effect sizes.

### Prediction of RTW 12 months after rehabilitation

The bivariate logistic regressions revealed 5 significant (*p* < 0.05) predictors (PCS, DSST 2, 6MWD, FIS total and SPSH) for RTW 12 months after rehabilitation discharge (see Tables A6, A7, A8, A9, A10, A11, A12, A13, A14, A15, A16, A17 and A18 in Supplementary Material 1). After multivariate logistic regression analysis with forward selection, FIS total at T4 remained a significant predictor of RTW at T4 (see Table 3). The logistic regression model was statistically significant (χ² (1) = 18.259, *p* < 0.001), indicating that the model reliably distinguishes between individuals who are able to work and those who are not. The model explained 24.7% (Nagelkerke R² = 0,247) of the variance in RTW. For FIS total, the OR was 0.969, and the 95% CI ranged from 0.953 to 0.986 (*p* < 0.001). This finding indicates that for each one-unit increase in the FIS total score, the odds of RTW decrease by 3.1%. Thus, severe symptoms of fatigue, as indicated by higher FIS total scores, are risk factors for RTW 12 months after rehabilitation discharge.

**Table 3:** Logistic regression analysis with RTW as outcome variable at measurement time point T4. FIS total was measured at T4

	B	SE	*p*	OR	95% CI
Intercept	2.843	0.825	< 0.001	17,160	
FIS total	-0.031	0.009	< 0.001	0.969	0.953, 0.986

FIS – Fatigue Impact Scale

### Prediction of perceived ability to work 12 months after rehabilitation

Spearman correlation analyses revealed 7 significant correlations with WAI total at T4 (*p* < 0.05) (see Table 4). Consequently, the PCS, 6MWD, FIS total, HADS-D_Depression_, SPSH, DSST 1, and DSST 2 were included in multiple regression analyses.

**Table 4:** Spearman correlation matrix with potential predictors for multivariate regression analysis at measurement time point T4

	WAI total	BMI	Age	PCS	6MWD	FIS total	HADS-D_Depression_	SPSH	DSST 1	DSST 2	MVPA	PIA
WAI total	1.00											
BMI	-0.09											
Age	-0.08	0.04										
PCS	-0.35***	0.08	-0.12									
6MWD	0.46***	-0.36***	-0.21*	-0.33**								
FIS total	-0.58***	0.07	-0.13	0.62***	-0.37***							
HADS-D_Depression_	-0.42***	0.24*	0.09	0.45***	-0.42***	0.59***						
SPSH	0.52***	-0.11	-0.05	-0.51***	0.46***	-0.70***	-0.60***					
DSST 1	0.30**	-0.24*	-0.35***	-0.20*	0.42***	-0.27**	-0.35***	0.32**				
DSST 2	0.26**	-0.15	-0.32**	-0.13	0.45***	-0.34***	-0.34***	0.32**	0.56***			
MVPA	0.20	0.01	-0.07	0.03	0.04	-0.13	-0.14	0.23*	-0.14	0.08		
PIA	0.09	-0.06	0.13	0.01	0.10	-0.10	-0.08	0.10	0.11	0.02	-0.43***	1.00

* *p* ≤ 0.05, ** *p* ≤ 0.01, *** *p* < 0.001; WAI – Work ability index, BMI – Body-Mass-Index, PCS - Post-Covid Syndrome, 6MWD – six-minute-walking-distance, FIS - Fatigue Impact Scale, HADS-D_Depression_ - Hospital Anxiety and Depression Scale (subscale depression), SPSH - subjective perceived status of physical and mental health, DSST - Digit Symbol Substitution Test, MVPA - moderate to vigorous activity, PIA - physical inactivity

Multiple regression analysis with stepwise integration revealed that the FIS total, the 6MWD, and the SPSH were significant predictors of the WAI total at T4 (see Table 5). The regression model was statistically significant (F(3,86) = 24.630, *p* < 0.001). The adjusted R² was 0.473, accounting for 47.3% of the variance in WAI total. FIS total was a significant predictor of the WAI total at T4. It had a regression standardized β of -0.399 (SE = 0.018, 95% CI = -0.100– -0.029). The negative coefficient indicates that higher FIS total scores are associated with a decrease in WAI total. The second significant predictor was the 6MWD with β = 0.196 (SE = 0.005, 95% CI = 0.001–0.023). The positive coefficient indicates that greater 6MWD is associated with an increase in the WAI total at T4. The SPSH had a standardized β coefficient of 0.237 (SE = 0.223, 95% CI = 0.009–0.894). A better perception of one’s own health (physical and mental) is associated with increased values in WAI total at T4.

**Table 5:** Multiple regression analysis with WAI total (T4) as outcome variable at measurement time point T4. The predictors were measured at T4

	b	SE	β	T	*p*	95% CI	Tolerance	VIF
Intercept	16.438	4.406		3.731	< 0.001	7.680, 25.196		
FIS total	-0.065	0.018	-0.399	-3.574	< 0.001	-0.100, -0.029	0.476	2.102
6MWD	0.012	0.005	0.196	2.251	0.027	0.001, 0.023	0.783	1.277
SPSH	0.451	0.223	0.237	2.027	0.046	0.009, 0.894	0.432	2.313

FIS – Fatigue Impact Scale, 6MWD – six-minute-walking-distance, SPSH - subjective perceived status of physical and mental health

**Fig. 1:**
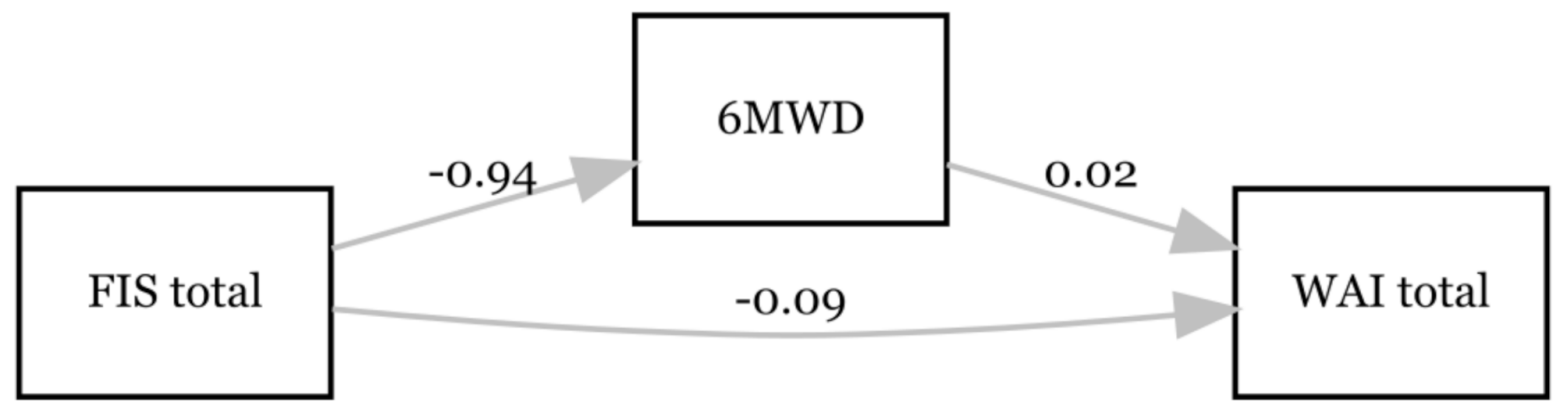
Associations between fatigue (FIS total), physical capacity (6MWD) and perceived work ability (WAI total) at T4. Only the significant paths are labelled with the β-coefficient. *N* = 109

To deepen the understanding of the underlying relationships between work ability (WAI total at T4) and the detected predictors (Table 5), the results of the mediation analyses are presented in the next step. The first mediation analysis was conducted with FIS total as a predictor, WAI total as an outcome variable and the 6MWD as a mediator (see Fig. 1). FIS total exerted a significant effect on 6MWD (β = -0.942; *p* < 0.001; 95% CI = -1.449– -0.436), which, in turn, demonstrated a significant effect on WAI total (β = 0.016; *p* = 0.003; 95% CI = 0.006–0.026). Furthermore, there was a significant direct effect of FIS total on WAI total (β = -0.088; *p* < 0.001; 95% CI=-0.144– -0.061). The bootstrap CI for the indirect effect excluded zero (β=-0.015, SE = 0.007, 95% CI=-0.031– -0.003). Accordingly, the results show a partial mediating effect of the 6MWD between the FIS total and the WAI total. The total model explained 40.7% of the variance in WAI total (R^2^ = 0.407; *p* < 0.001).

**Fig. 2:**
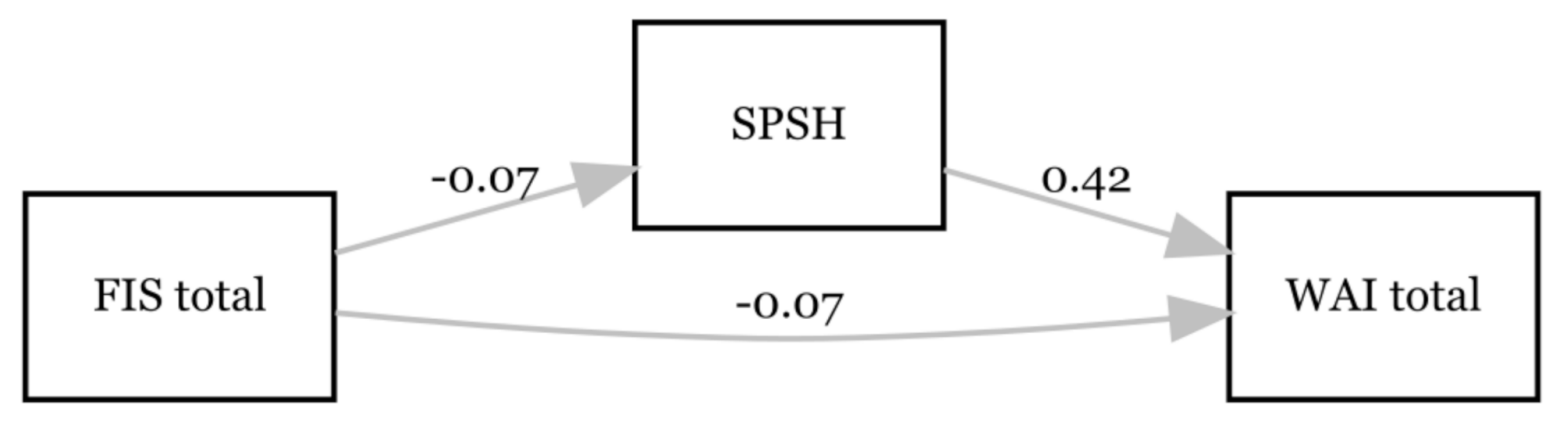
Associations between fatigue (FIS total), subjective perceived status of physical and mental health (SPSH) and perceived work ability (WAI total) at T4. Only the significant paths are labelled with the β-coefficient. *N* = 109

A second mediation analysis was conducted with FIS total as predictor, WAI total as an outcome variable and the SPSH (see Fig. 2). The FIS total significantly affected SPSH (β = -0.065; *p* < 0.001; 95% CI = -0.077– -0.053), which, in turn, demonstrated a significant effect on WAI total (β = 0.416; *p* = 0.030; 95% CI = 0.040–0.792). Furthermore, there was a significant direct effect of FIS total on WAI total (β = -0.074; *p* < 0.001; 95% CI = -0.108– -0.040). The bootstrap CI for the indirect effect excluded zero (β = -0.027, SE = 0.013, 95% CI = -0.052– -0.001). Accordingly, the results show a partial mediation effect of SPSH between FIS total and WAI total. The total model explained 39.6% of the variance in WAI total (R^2^ = 0.396; *p* < 0.001).

**Fig. 3:**
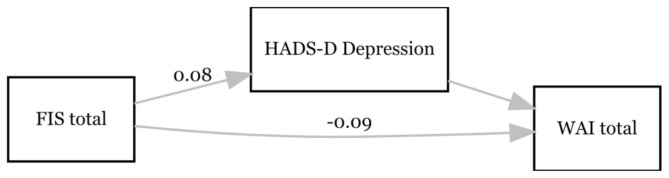
Associations between fatigue (FIS total), depression (HADS-D_Depression_) and perceived work ability (WAI total) at T4. Only the significant paths are labelled with the β-coefficient. *N* = 102

A third mediation analysis was conducted with FIS total as a predictor, WAI total as an outcome variable and depression as mediator (see Fig. 3). FIS total significantly affected HADS-D_Depression_ (β = 0.077; *p* < 0.001; 95% CI = 0.056–0.098). The effect of the HADS-D_Depression_ on the WAI total was not significant (β = -0.111; *p* > 0.05; 95% CI = -0.343–0.120). Furthermore, there was a significant direct effect of FIS total on WAI total (β = -0.092; *p* < 0.001; 95% CI = -0.122– -0.061). The bootstrap CI for the indirect effect included zero (β = -0.009, SE = 0.009, 95% CI = -0.025– -0.012). Accordingly, the results revealed no mediating effect of HADS-D_Depression_ between FIS total and the WAI total. The combined influence of FIS total and HADS-D_Depression_ explained 40.0% of the variance of WAI total (R^2^ = 0.400; *p* < 0.001).

**Fig. 4:**
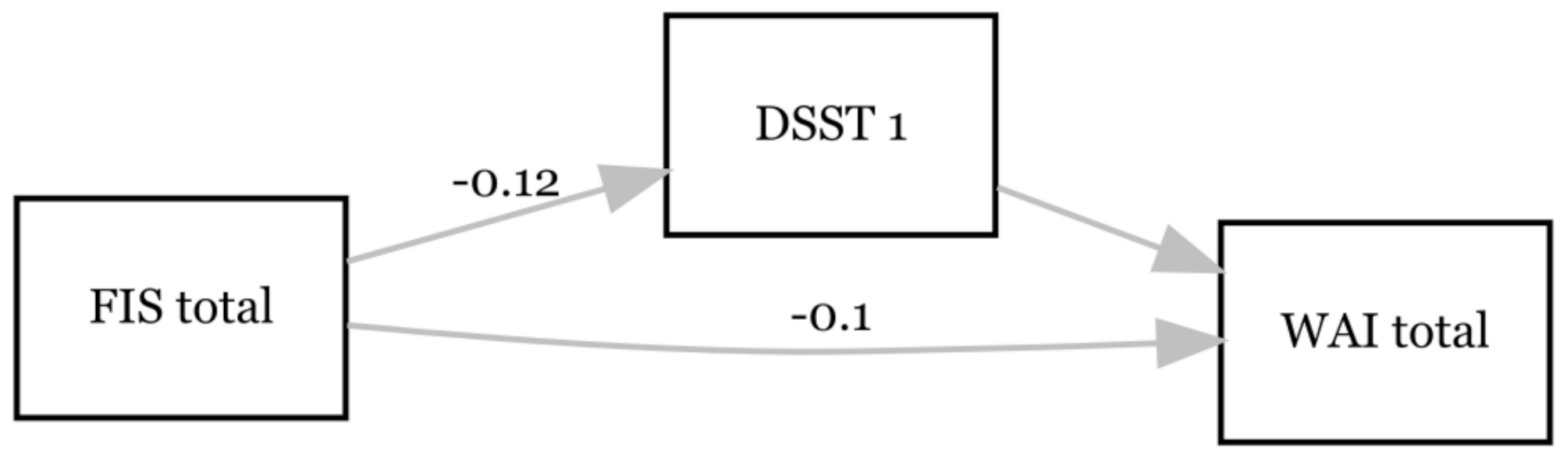
Associations between fatigue (FIS total), cognition (DSST 1) and perceived work ability (WAI total) at T4. Only the significant paths are labelled with the β-coefficient. *N* = 95

Finally, FIS total exerted a significant effect on DSST1 (β = -0.121; *p* = 0.003; 95% CI = -0.202– -0.041) (see Fig. 4). The effect of DSST1 on WAI total was not significant (β = -0.049; *p* > 0.05; 95% CI = -0.015–0.114). Furthermore, there was a significant effect of FIS total on WAI total (β = -0.099; *p* < 0.001; 95% CI = -0.125– -0.072). The bootstrap CI for the indirect effect included zero (β = -0.006, SE = 0.005, 95% CI = -0.017– -0.002). Accordingly, the results show no mediating effect of DSST1 between FIS total and WAI total. The combined influence of FIS total and DSST1 explained 43.0% of the variance of WAI total (R^2^ = 0.430; *p* < 0.001).

## Discussion

This study provides valuable insights into the long-term outcomes of the work ability of post-COVID patients 12 months after rehabilitation. In addition, the results revealed physical and neuropsychological (risk-)factors influencing work ability and RTW in the long-term and their underlying relationships.

### Changes in work ability and the subjective prognosis of employment over time

Twelve months after rehabilitation program, the WAI total (Mdn = 21) indicated a poor work ability and was significantly worse than at T1. A differentiated analysis of the seven sub-dimensions of the WAI shows that mainly three aspects lead to this deterioration. Some patients suffer other chronic diseases after SARS-CoV-2 infection, which may further impair their work ability (Dimension 3). Second, the results of Dimension 4 demonstrate an increase in the patient’s estimated loss of work ability due to illness. Finally, the worker’s own prognosis of future work ability is worsening as well. One reason for the poor work ability may be the continued high prevalence of post-COVID symptoms in this study population, as reported in Müller et al. [36]. Nielsen et al. [55] also confirmed a decrease in work ability in patients with persistent post-COVID symptoms 12 months after infection. Compared with studies, the perceived ability to work of post-COVID patients in our study was lower [56, 57]. Rutsch and Deck [57] assessed a mean score of 5.9 on the WAI Dimension 1 12 months after rehabilitation, which was almost twice as high as that reported in our population at T4. Furthermore, compared with prior rehabilitation Rutsch and Deck [57] could lead to an improvement in the ability to work over time. This discrepancy may be attributed to the high number of healthcare workers in our study, a population whose biopsychosocial health has been negatively affected by the COVID-19 pandemic due to changes in working condition [58, 59]. Additionally, the long duration since acute COVID-19 may also contribute to these differences. The results of the SPE scale over time show that the patients perceive their prognosis of gainful employment to be slightly lower 12 months after rehabilitation than at T1. A significant difference was observed. This is in line with the results of the WAI total. Rutsch and Deck [57] assessed the SPE scale 12 months after rehabilitation as well and did not find a significant difference within the 12-month course. Only 48,6% of post-COVID patients returned to work 12 months after rehabilitation in the current study. This rate is consistent with Delgado-Alonso et al. [14], who reported approximately the same prevalence (49.4%) 20.71 (SD: 6.50) months after clinical onset. On the other hand, this prevalence is less than the prevalence of 60.9% in the review of Ottiger et al. [60] with 21.155 post-COVID patients, suggesting that the characteristics of our participants may have contributed to the lower RTW rate. However, our findings reflect the broader challenges associated with RTW in post-COVID patients. Importantly, patients who returned to work may experience a decline in their quality of life, which may in turn result in a reduction in their work productivity [56]. Within our study, patients receiving psychological treatment after rehabilitation show a worse trend in absence days (WAI Dimension 5) compared to those without psychological treatment, highlighting the complex interaction between mental health and work ability. Financial losses from extended absences can be unsustainable for the patients, leading to psychological issues such as depression, financial instability, and even existential fears [61]. In addition to individual post-COVID symptoms several work ability obstacles are mentioned in the literature: lack of control over work pressures, inappropriate policies for the management of sickness absence, and COVID-compliant organisational cultures [31, 62]. In general, only a few studies have examined the long-term impact of post-COVID on work ability and RTW.

The current results indicate that measures to restore the patient’s ability to work are urgently needed as part of the aftercare process. Our findings suggest that personalized interventions should include gradually introducing patients to the required workload and teaching them suitable self-management strategies (e.g., the PACING technique, recognition their own tolerance thresholds, and organisation of working time). Additionally, structural factors (e.g., availability of workplace adaptations, supportive policies, and vocational rehabilitation programs) within long-term and regularly reviewed plans for RTW should be adapted to the needs of the patient groups [57, 60, 62]. Due to the very diverse symptom cluster of post-COVID patients it may be necessary to enable more flexible working hours, provide a working environment that responds to the needs of the patients and to adapt working task individually to the patients [22, 62]. Furthermore, the European Agency for Safety and Health at work published guidelines with well-founded recommendations and procedures for the successful RTW of post-COVID patients for both employees and employers [63]. Educating staff and employers about the diverse course of post-COVID disease and its long-term effects is crucial to avoid stigmatising post-COVID patients during their RTW process. Stigmatisation can worsen the recovery of post-COVID patients and even prevent them from seeking adequate assistance [64, 65]. Early collaboration between health and accident insurers, rehabilitation and aftercare doctors and therapists, and employers is essential to ensure the optimal care and a successful RTW for these patients. For example, testing workplace-related activities during inpatient or outpatient rehabilitation to identify barriers could be possible [57]. Overall, our study emphasizes the importance of these interventions and collaborations to enhance the RTW process for post-COVID patients.

### Factors influencing work ability or RTW

Both conducted regression analyses confirmed factors influencing work ability 12 months after rehabilitation. The influencing factors differ regarding RTW or perceived ability to work.

In addition to fatigue (FIS total) the post-COVID severity (PCS), physical capacity (6MWD), cognitive function (DSST2) and the subjective perceived status of physical and mental health (SPSH) were significant predictors in separate bivariate logistic regression analyses. However, only the severity of fatigue primarily affected RTW in a long term in the comprehensive multivariate logistic regression, which included all five predictors. The FIS is a multidimensional construct that includes cognitive, physical, and psychosocial functioning. Therefore, fatigue may account for the variance explained by the other (unidimensional) predictors, e.g., 6MWD or DSST2. Our results show that each one-unit increase in the FIS total score, the odds of being able to work 12 months after inpatient rehabilitation decrease by 3.1%. This finding is supported by the results of previous studies. Green et al. [18] and Diem et al. [13] reported higher fatigue rates in post-COVID patients who were unable to work than in those who were working and Aben et al. [28] revealed that symptoms of fatigue are strongly associated with time to RTW after SARS-CoV-2 infection. In general, fatigue is one of the most reported post-COVID symptom and the current literature shows, that even more than two years after acute infection the prevalence of fatigue is between 44 and 60% [66–68]. The results of our own study revealed that the severity of fatigue worsened even 12 months after rehabilitation [69]. Given these challenges, the treatment of fatigue is a crucial element in ensuring patients’ long-term occupational and social participation. On the basis of Greenhalgh et al. [67] and Weise et al. [70], the following comprehensive approaches to managing fatigue in daily life can be summarized: multidisciplinary care (e.g., with doctors from different disciplines, occupational therapists, psychological staff, social workers), attention to self-management according to prioritizing, planning, pacing to increase exercise tolerance and to avoid post exertional malaise (PEM) and the education of patients to have a full understanding of fatigue. Furthermore, gradual RTW plans with appropriate adjustments have shown positive long-term effects on work-related fatigue, overall functioning, and work ability as well as RTW [71].

When focusing on the perceived ability to work (WAI total) post-COVID severity (PCS), physical capacity (6MWD), cognitive function (DSST1, DSST2), depression (HADS-D_Depression_), and fatigue (FIS total) are considered. As shown by the multiple regression analysis the severity of fatigue is an important predictor of perceived ability to work. In this context, however, fatigue is a significant predictor together with physical capacity (6MWD) and the SPSH. Braig et al. [21] demonstrated that post-COVID patients with fatigue have a greater risk of low work ability, while Hasenoehrl et al. [72] revealed a significant association between physical capacity and work ability in healthcare workers after an exercise intervention. Fatigue also seems to influence work ability in patients with other diseases, e.g., breast cancer and multiple sclerosis [73, 74]. In general, fatigue may influence post-COVID patients’ work ability in part because of alterations in focused and sustained attention [75]. The conducted mediation analysis further revealed that the association between fatigue severity and work ability is mediated by both physical capacity and SPSH. These findings suggest that the improvement of patients’ physical capacity e.g., through individualized physical exercise training, considering the presence of PEM, and the strengthening of self-awareness of one’s own health e.g., through mediations or body scan methods, may mitigate the negative consequences of fatigue on patients’ ability to work. After an eight-week exercise intervention Hasenoehrl et al. [72] showed significant improvements in physical capacity, psychological health as well as work ability. Although a significant direct effect of fatigue on cognitive function and depression was shown in the preset study, the association between fatigue severity and work ability was not mediated by depression or cognitive function, contrary to previously described results. Greenhalgh et al. [67] mentioned in their clinical update for long COVID potential links between fatigue and cognitive function. The link between symptoms of fatigue and depression was demonstrated previously by Badinlou et al. [76] and Teopiz et al. [77]. Higher fatigue scores were positively correlated with higher scores of depression, with a medium to high effect.

## Limitations

When interpreting the current results some limitations need to be considered. The use of a longitudinal observational study design without a control group means that the current results must be treated with caution. The relatively small sample size, which included only one hospital specializing in occupational diseases, must be taken into account when applying statistical methods. In addition, the generalizability of the results should be considered against the background that our study focused on a specific study population with work-related SARS-CoV-2 infection. Furthermore, in our study population the alpha and delta variants were predominant with a higher incidence of post-COVID symptoms compared to the omicron variant [78]. Persistent post-COVID symptoms are associated with worsening work ability, which suggests that later variants may have less impact on work ability and RTW [28]. In particular, fatigue seems to be a relevant predictor of work ability. Additionally, significant aspects of occupational reintegration, such as vocational training, occupational rehabilitation, and socio-medical performance assessments, were not included in our study. The absence of this information limits our understanding of the full scope of the reintegration process and its influence on long-term outcomes. This should be analysed more consistently in future studies (e.g., as part of individual case studies focusing on the process of reintegration of post-COVID patients).

## Conclusions

The current results revealed limited work ability and a high prevalence of post-COVID patients who did not return to work 12 months after rehabilitation. In particular, fatigue seems to be an important predictor of work ability and RTW in these patients. Therefore, it is necessary to assess and monitor fatigue. The chances of full recovery in patients who have suffered from post-COVID for 2 years or more appear to be low [66]. These circumstances can affect the ability to work and, as a result, the biopsychosocial health and health-related quality of life for a long period of time [79–81]. Individualized, tailored, and targeted interventions need to be developed and implemented in the rehabilitation process to contribute to disease recovery as well as the biopsychosocial health and work ability of post-COVID patients.

## Electronic supplementary material

Below is the link to the electronic supplementary material.

Supplementary Material 1

Supplementary Material 2

## Data Availability

The data are available from the corresponding author upon request.

## Abbreviations

BMI: Body-Mass-Index
COVID-19: Coronavirus disease 2019
CI: Confidence Intervall
DRKS: German Register for Clinical Studies
DSST: Digit Symbol Substitution Test
FIS: Fatigue Impact Scale
HADS-D_Depression_: German version of the Hospital Anxiety and Depression Scale (subscale depression)
IQR: Interquartile range
M: Mean
Mdn: Median
MET: Metabolic equivalent of task
MVPA: Moderate to vigorous physical activity
PA: Physical activity
PCS: Post-Covid Syndrome
PEM: Postexertional malaise
PIA: Physical inactivity
RTW: Return to work
SARS-CoV-2: Severe acute respiratory syndrome coronavirus type 2
6MWD: Six-minute-walking-distance
SES: Socioeconomic status
SPE: Subjective prognosis of employment
SPSH: Subjective perceived status of physical and mental health
VIF: Variance inflation factor
WAI: Work ability index
