# Supplementary material for: Long-term course and factors influencing work ability and return to work in post-COVID patients 12 months after inpatient rehabilitation
Source: J Occup Med Toxicol. 2024 Nov 1;19:43. doi: 10.1186/s12995-024-00443-4 (PMC11529184; doi:10.1186/s12995-024-00443-4)
Supplement: Supplementary file 1 — Supplementary Material 1 [file 12995_2024_443_MOESM1_ESM.docx]

**Additional file 1**

| **Table A1:** Groupwise comparison of perceived work ability and subjective prognosis of gainful employment (SPE) of patients undergoing psychotherapeutic treatment and patients without psychotherapeutic treatment between timepoints T1 and T4. | | | | | | | | | | | |
| --- | --- | --- | --- | --- | --- | --- | --- | --- | --- | --- | --- |
|  | **Outpatient psychological treatment (YES)** | | | | **Outpatient psychological treatment (NO)** | | | | **Between-group**  **difference** | | |
|  | **N** | **T1**  **Median**  **(IQR)** | **T4**  **Median**  **(IQR)** | **Δ** | **N** | **T1**  **Median**  **(IQR)** | **T4**  **Median**  **(IQR)** | **Δ** | **z** | **p** | **r** |
| WAI Dimension 1 (0-10) | 53 | 2.00  (0.00-4.00) | 0.00  (0.00-3.50) | 0.00  (-2.00-1.00) | 55 | 5.00  (2.00-6.00) | 5.00  (3.00-6.00) | 1.00  (-1.00-2.00) | 1.192 | 0.233 | 0.115 |
| WAI Dimension 2 (2-10) | 53 | 8.00  (7.00-9.00) | 8.00  (6.00-9.00) | 0.00  (-1.00-0.75) | 55 | 6.50  (5.00-8.00) | 6.00  (5.00-7.50) | 0.00  (-1.00-1.00) | 0.287 | 0.774 | 0.028 |
| WAI Dimension 3  (1-7) | 54 | 2.00  (2.00-3.00) | 2.00  (2.00-2.00) | 0.00  (-0.25-0.00) | 55 | 2.00  (2.00-3.00) | 2.00  (2.00-2.00) | 0.00  (-1.00-0.00) | -0.073 | 0.942 | 0.007 |
| WAI Dimension 4 (1-6) | 53 | 3.00  (1.00-4.00) | 2.00  (1.00-3.00) | -1.00  (-2.00-0.00) | 54 | 4.00  (3.00-5.00) | 3.00  (2.00-4.25) | 0.00  (-2.00-0.00) | 0.846 | 0.398 | 0.082 |
| WAI Dimension 5 (1-5) | 53 | 1.00  (1.00-2.00) | 1.00  (1.00-2.00) | 0.00  (-1.00-0.00) | 55 | 2.00  (1.00-3.00) | 2.00  (1.00-3.00) | 0.00  (-1.00-1.00) | 2.206 | 0.027 | 0.213 |
| WAI Dimension 6 (1-7) | 53 | 4.00  (1.00-4.00) | 4.00  (1.00-4.00) | 0.00  (-3.00-0.00) | 53 | 4.00  (4.00-7.00) | 4.00  (4.00-4.00) | 0.00  (-3.00-0.00) | -0.902 | 0.367 | -0.088 |
| WAI Dimension 7 (1-4) | 53 | 2.00  (1.00-2.50) | 2.00  (1.00-2.00) | 0.00  (0.00-0.50) | 55 | 2.00  (2.00-3.00) | 2.00  (2.00-3.00) | 0.00  (-1.00-0.00) | -0.531 | 0.596 | 0.051 |
| WAI Total  (7-49) | 53 | 23.00  (20.00-26.00) | 18.50  (16.00-23.00) | -3.00  (-6.00-0.50) | 53 | 30.00  (22.50-30.00) | 25.00  (20.00-28.50) | -2.00  (-3.50-1.00) | 1.701 | 0.089 | 0.165 |
| SPE scale  (0-3) | 53 | 2.00  (2.00-3.00) | 3.00  (2.00-3.00) | 0.00  (0.00-1.00) | 54 | 2.00  (1.00-2.00) | 2.00  (1.00-3.00) | 0.00  (0.00-1.00) | 0.061 | 0.952 | 0.006 |
| WAI – Work ability index, SPE – Subjective prognosis of gainful employment, IQR – Interquartile range. | | | | | | | | | | | |

| **Table A2:** Groupwise comparison of perceived work ability and subjective prognosis of gainful employment (SPE) of patients undergoing ambulatory active physiotherapeutic treatment and patients without ambulatory active physiotherapeutic treatment between timepoints T1 and T4. | | | | | | | | | | | |
| --- | --- | --- | --- | --- | --- | --- | --- | --- | --- | --- | --- |
|  | **Ambulatory active physiotherapeutic treatment (YES)** | | | | **Ambulatory active physiotherapeutic treatment (NO)** | | | | **Between-group**  **difference** | | |
|  | **N** | **T1**  **Median**  **(IQR)** | **T4**  **Median**  **(IQR)** | **Δ** | **N** | **T1**  **Median**  **(IQR)** | **T4**  **Median**  **(IQR)** | **Δ** | **z** | **p** | **r** |
| WAI Dimension 1 (0-10) | 88 | 24.00  (20.63-27.00) | 21.00  (17.00-25.87) | -3.00  (-4.87-0.75) | 20 | 4.50  (1.23-5.75) | 4.50  (0.00-7.75) | 0.00  (-1.00-2.00) | -0.435 | 0.664 | -0.041 |
| WAI Dimension 2 (2-10) | 90 | 3.00  (0.75-5.00) | 3.00  (0.00-5.25) | 0.00  (-2.00-1.25) | 20 | 6.25  (5.62-8.75) | 6.00  (5.00-8.75) | 0.00  (-1.00-1.00) | -0.531 | 0.595 | -0.051 |
| WAI Dimension 3  (1-7) | 91 | 2.00  (2.00-3.00) | 2.00  (2.00-2.00) | 0.00  (0.00-0.00) | 20 | 2.00  (2.00-4.75) | 2.00  (2.00-2.00) | 0.00  (-1.75-0.00) | 0.932 | 0.351 | 0.088 |
| WAI Dimension 4 (1-6) | 89 | 3.00  (2.00-4.00) | 2.00  (1.00-3.00) | 0.00  (-2.00-0.00) | 20 | 4.00  (3.00-6.00) | 3.00  (1.00-5.00) | -0.50  (-2.50-0.00) | 0.093 | 0.926 | 0.009 |
| WAI Dimension 5 (1-5) | 90 | 2.00  (1.00-2.00) | 1.00  (1.00-2.00) | 0.00  (-1.00-0.00) | 20 | 2.00  (1.00-4.75) | 2.50  (1.00-4.00) | 0.00  (-0.75-1.00) | -0.791 | 0.429 | -0.075 |
| WAI Dimension 6 (1-7) | 88 | 4.00  (4.00-4.00) | 4.00  (1.00-4.00) | 0.00  (-3.00-0.00) | 20 | 4.00  (4.00-6.25) | 4.00  (4.00-4.00) | 0.00  (-2.25-0.00) | -0.387 | 0.699 | -0.031 |
| WAI Dimension 7 (1-4) | 90 | 2.00  (2.00-3.00) | 2.00  (2.00-3.00) | 0.00  (-0.25-0.00) | 20 | 2.00  (1.00-3.00) | 3.00  (2.00-3.00) | 0.00  (0.00-1.00) | -1.625 | 0.104 | -0.155 |
| WAI Total  (7-49) | 88 | 24.00  (20.63-27.00) | 21.00  (17.00-25.87) | -3.00  (-4.87-0.75) | 20 | 26.00  (22.25-31.50) | 26.50  (17.62-30.87) | -3.00  (-5.00-0.75) | -0.012 | 0.990 | -0.001 |
| SPE scale  (0-3) | 89 | 2.00  (1.00-3.00) | 2.00  (1.00-3.00) | 0.00  (0.00-1.00) | 20 | 2.00  (1.00-2.75) | 2.00  (1.00-3.00) | 0.00  (0.00-0.00) | 1.014 | 0.311 | 0.097 |
| WAI – Work ability index, SPE – Subjective prognosis of gainful employment, IQR – Interquartile range. | | | | | | | | | | | |

| **Table A3:** Groupwise comparison of perceived work ability and subjective prognosis of gainful employment (SPE) of patients undergoing repeated rehabilitation and patients without repeated rehabilitation between timepoints T1 and T4. | | | | | | | | | | | |
| --- | --- | --- | --- | --- | --- | --- | --- | --- | --- | --- | --- |
|  | **Repeated rehabilitation (YES)** | | | | **Repeated rehabilitation (NO)** | | | | **Between-group**  **difference** | | |
|  | **N** | **T1**  **Median**  **(IQR)** | **T4**  **Median**  **(IQR)** | **Δ** | **N** | **T1**  **Median**  **(IQR)** | **T4**  **Median**  **(IQR)** | **Δ** | **z** | **p** | **r** |
| WAI Dimension 1 (0-10) | 28 | 2.00  (0.25-4.00) | 0.00  (0.00-4.75) | 0.00  (-2.00-2.009 | 82 | 4.00  (1.00-5.00) | 3.00  (0.00-6.00) | 0.00  (-1.00-1.25) | 0.177 | 0.860 | 0.017 |
| WAI Dimension 2 (2-10) | 28 | 8.00  (6.62-9.00) | 7.25  (6.00-8.37) | 0.00  (-1.00-0.00) | 82 | 7.00  (6.00-8.25) | 7.00  (6.00-8.00) | 0.00  (-1.00-1.00) | 0.604 | 0.546 | 0.058 |
| WAI Dimension 3  (1-7) | 28 | 2.00  (2.00-2.00) | 2.00  (2.00-2.00) | 0.00  (0.00-0.00) | 82 | 2.00  (2.00-3.00) | 2.00  (2.00-2.00) | 0.00  (-1.00-0.00) | -0.366 | 0.715 | -0.035 |
| WAI Dimension 4 (1-6) | 28 | 4.00  (2.00-5.00) | 2.00  (1.00-3.00) | -1.00  (-3.75-0.00) | 81 | 3.00  (2.00-4.00) | 2.00  (1.00-4.00) | 0.00  (-2.00-0.00) | 1.592 | 0.111 | 0.152 |
| WAI Dimension 5 (1-5) | 28 | 2.00  (1.00-2.00) | 1.00  (1.00-2.00) | 0.00  (-1.00-0.00) | 82 | 2.00  (1.00-2.25) | 2.00  (1.00-3.00) | 0.00  (-1.00-0.00) | 1.260 | 0.208 | 0.120 |
| WAI Dimension 6 (1-7) | 28 | 4.00  (4.00-4.00) | 4.00  (1.00-4.00) | 0.00  (0.00-0.00) | 80 | 4.00  (4.00-4.00) | 4.00  (1.00-4.00) | 0.00  (-3.00-0.00) | -1.659 | 0.097 | -0,160 |
| WAI Dimension 7 (1-4) | 28 | 2.00  (1.00-3.00) | 2.00  (2.00-2.00) | 0.00  (0.00-0.00) | 82 | 2.00  (2.00-3.00) | 2.00  (2.00-3.00) | 0.00  (-1.00-1.00) | 0.397 | 0.691 | 0.038 |
| WAI Total  (7-49) | 28 | 24.00  (20.63-26.75) | 20.00  (16.12-25.00) | -3.00  (-5.62-0.00) | 80 | 25.00  (21.00-28.00) | 22.00  (18.00-27.00) | -3.00  (-5.00-1.00) | 0.655 | 0.513 | 0,063 |
| SPE scale  (0-3) | 28 | 2.00  (1.25-3.00) | 2.00  (1.00-3.00) | 0.00  (-0.75-1.00) | 81 | 2.00  (1.00-3.00) | 2.00  (1.00-3.00) | 0.00  (0.00-1.00) | 1.095 | 0.274 | 0.105 |
| WAI – Work ability index, SPE – Subjective prognosis of gainful employment, IQR – Interquartile range. | | | | | | | | | | | |

| **Table A4:** Groupwise comparison of perceived work ability and subjective prognosis of gainful employment (SPE) of patients who exercised in an outpatient group and patients who did not exercise in an outpatient group between timepoints T1 and T4. | | | | | | | | | | | |
| --- | --- | --- | --- | --- | --- | --- | --- | --- | --- | --- | --- |
|  | **Exercising in an outpatient group (YES)** | | | | **Exercising in an outpatient group (NO)** | | | | **Between-group**  **difference** | | |
|  | **N** | **T1**  **Median**  **(IQR)** | **T4**  **Median**  **(IQR)** | **Δ** | **N** | **T1**  **Median**  **(IQR)** | **T4**  **Median**  **(IQR)** | **Δ** | **z** | **p** | **r** |
| WAI Dimension 1 (0-10) | 18 | 2.00  (0.00-4.25) | 0.00  (0.00-6.00) | 0.00  (-0.50-1.25) | 86 | 3.00  (1.00-5.00) | 3.00  (0.00-6.00) | 0.00  (-2.00-1.25) | -0.729 | 0.466 | -0.071 |
| WAI Dimension 2 (2-10) | 18 | 8.00  (7.00-9.00) | 8.00  (5.87-10.00) | 0.00  (-1.00-0.25) | 86 | 7.00  (6.00-9.00) | 7.00  (6.00-8.00) | 0.00  (-1.00-1.00) | 0.449 | 0.654 | 0.044 |
| WAI Dimension 3  (1-7) | 18 | 2.00  (2.00-2.25) | 2.00  (2.00-2.00) | 0.00  (-0.25-0.00) | 87 | 2.00  (2.00-3.00) | 2.00  (2.00-2.00) | 0.00  (-1.00-0.00) | -0.227 | 0.820 | -0.022 |
| WAI Dimension 4 (1-6) | 18 | 3.00  (1.75-4.25) | 1.50  (1.00-3.25) | -0.50  (-2.25-0.00) | 85 | 4.00  (2.00-5.00) | 2.00  (1.00-4.00) | -1.00  (-2.00-0.00) | -0.076 | 0.939 | 0.007 |
| WAI Dimension 5 (1-5) | 18 | 1.00  (1.00-2.25) | 1.00  (1.00-2.00) | 0.00  (-1.00-0.00) | 86 | 2.00  (1.00-2.00) | 1.50  (1.00-3.00) | 0.00  (-1.00-0.00) | 0.456 | 0.648 | 0.045 |
| WAI Dimension 6 (1-7) | 18 | 4.00  (1.00-4.00) | 1.00  (1.00-4.00) | 0.00  (-3.00-0.00) | 84 | 4.00  (4.00-4.00) | 4.00  (1.00-4.00) | 0.00  (-3.00-0.00) | 0.444 | 0.657 | 0.044 |
| WAI Dimension 7 (1-4) | 18 | 2.00  (1.75-3.00) | 2.00  (1.75-3.00) | 0.00  (-0.25-0.00) | 86 | 2.00  (2.00-3.00) | 2.00  (2.00-3.00) | 0.00  (0.00-0.25) | 0.546 | 0.585 | 0.054 |
| WAI Total  (7-49) | 18 | 21.50  (19.00-28.00) | 18.50  (165.00-23.25) | -3.00  (-4.25-0.00) | 84 | 25.00  (2.00-28.00) | 22.00  (18.00-27.00) | -3.00  (-5.00-1.00) | 0.057 | 0.954 | 0.006 |
| SPE scale  (0-3) | 18 | 2.00  (0.00-3.00) | 3.00  (0.75-3.00) | 0.00  (0.00-0.00) | 85 | 2.00  (1.00-2.00) | 2.00  (1.00-3.00) | 0.00  (0.00-1.00) | 0.614 | 0.539 | 0.060 |
| WAI – Work ability index, SPE – Subjective prognosis of gainful employment, IQR – Interquartile range. | | | | | | | | | | | |

| **Table A5:** Descriptive statistics of potential predictors of perceived workability and RTW 12 months after rehabilitation discharge (T4) | | | | |
| --- | --- | --- | --- | --- |
|  | **N** | **Min** | **Max** | **Median**  **(IQR)** |
| PCS | 113 | 0.00 | 55.50 | 40.00  (32.50 - 48.50) |
| 6MWD | 95 | 330.00 | 852.00 | 571.00  (502.00 - 628.00) |
| FIS total | 114 | 0.00 | 159.00 | 97.00  (72.50 - 117.00) |
| HADS-D_Depression_ | 105 | 0.00 | 20.00 | 6.00  (4.00 - 11.00) |
| SPSH | 111 | 1.59 | 18.99 | 10.77  (8.40 - 12.64) |
| DSST 1 | 98 | 13.00 | 88.00 | 49.00  (37.75 - 58.25) |
| DSST 2 | 98 | 0 | 9 | 6.00  (4.00 - 8.00) |
| MVPA | 83 | 11.83 | 182.07 | 47.51  (33.25 - 69.99) |
| PIA | 83 | 7.83 | 18.58 | 14.14  (12.97 - 15.60) |
| PCS - Post-Covid Syndrome, 6MWD – six-minute-walking-distance, FIS - Fatigue Impact Scale, HADS-D_Depression_ - Hospital Anxiety and Depression Scale (subscale depression), SPSH - subjective perceived status of physical and mental health, DSST - Digit Symbol Substitution Test, MVPA - moderate to vigorous activity, PIA - physical inactivity, IQR – Interquartile Range | | | | |

| **Table A6:** Bivariate logistic regression analyses with RTW (yes/no) (T4) as outcome variable and sex as predictor | | | | | |
| --- | --- | --- | --- | --- | --- |
| Predictor | B | SE | p | OR | 95% CI |
| Intercept | 0.426 | 0.816 | 0.602 | 1.531 | 0.308-7.829 |
| Sex | -0.272 | 0.449 | 0.545 | 0.762 | 0.312-1.839 |
| Nagelkerkes R^2^ =0.004 | | | | | |

| **Table A7:** Bivariate logistic regression analyses with RTW (yes/no) (T4) as outcome variable and BMI as predictor | | | | | |
| --- | --- | --- | --- | --- | --- |
| Predictor |  | SE | p | OR | 95% CI |
| Intercept | -1.605 | 1.008 | 0.111 | 0.201 | 0.026-1.391 |
| BMI | 0.049 | 0.032 | 0.118 | 1.051 | 0.989-1.121 |
| Nagelkerkes R^2^ =0.030 | | | | | |

| **Table A8:** Bivariate logistic regression analyses with RTW (yes/no) (T4) as outcome variable and age as predictor | | | | | |
| --- | --- | --- | --- | --- | --- |
| Predictor | B | SE | p | OR | 95% CI |
| Intercept | -0.526 | 0.916 | 0.566 | 0.591 | 0.094-3.540 |
| Age | 0.009 | 0.018 | 0.598 | 1.009 | 0.975-1.046 |
| Nagelkerkes R^2^ =0.003 | | | | | |

| **Table A9:** Bivariate logistic regression analyses with RTW (yes/no) (T4) as outcome variable and PCS Score (T4) as predictor | | | | | |
| --- | --- | --- | --- | --- | --- |
| Predictor | B | SE | p | OR | 95% CI |
| Intercept | 1.762 | 0.765 | 0.021 | 5.825 | 1.412-29.127 |
| PCS Score | -0.045 | 0.018 | 0.014 | 0.956 | 0.919-0.989 |
| Nagelkerkes R^2^ =0.095 | | | | | |
| PCS - Post-Covid Syndrome | | | | | |

| **Table A10:** Bivariate logistic regression analyses with RTW (yes/no) (T4) as outcome variable and COVID status as predictor | | | | | |
| --- | --- | --- | --- | --- | --- |
| Predictor | B | SE | p | OR | 95% CI |
| Intercept | -0.501 | 0.579 | 0.387 | 0.606 | 0.193-1.881 |
| COVID-19 severity | 0.354 | 0.434 | 0.415 | 1.425 | 0.610-3.381 |
| Nagelkerkes R^2^ =0.008 | | | | | |
| COVID-19 severity - 0-mild/moderate, 1-severe/critical | | | | | |

| **Table A11:** Bivariate logistic regression analyses with RTW (yes/no) (T4) as outcome variable and HADS-D_Depression_ (T4) as predictor | | | | | |
| --- | --- | --- | --- | --- | --- |
| Predictor | B | SE | p | OR | 95% CI |
| Intercept | 0.524 | 0.372 | 0.159 | 1.689 | 0.822-3.571 |
| HADS-D_Depression_ | -0.078 | 0.045 | 0.085 | 0.925 | 0.844-1.009 |
| Nagelkerkes R^2^ =0.040 | | | | | |
| HADS-D_Depression_ - Hospital Anxiety and Depression Scale (subscale depression) | | | | | |

| **Table A12:** Bivariate logistic regression analyses with RTW (yes/no) (T4) as outcome variable and FIS total (T4) as predictor | | | | | |
| --- | --- | --- | --- | --- | --- |
| Predictor | B | SE | p | OR | 95% CI |
| Intercept | 2.347 | 0.708 | 0.001 | 10.451 | 2.859-46.741 |
| FIS total | -0.026 | 0.007 | <0.001 | 0.975 | 0.960-0.988 |
| Nagelkerkes R^2^ =0.182 | | | | | |
| FIS - Fatigue Impact Scale | | | | | |

| **Table A13:** Bivariate logistic regression analyses with RTW (yes/no) (T4) as outcome variable and 6MWD (T4) as predictor | | | | | |
| --- | --- | --- | --- | --- | --- |
| Predictor | B | SE | p | OR | 95% CI |
| Intercept | -3.330 | 1.441 | 0.021 | 0.036 | 0.002-0.536 |
| 6MWD | 0.006 | 0.003 | 0.019 | 1.006 | 1.001-1.011 |
| Nagelkerkes R^2^ =0.087 | | | | | |
| 6MWD - six-minute-walking-distance | | | | | |

| **Table A14:** Bivariate logistic regression analyses with RTW (yes/no) (T4) as outcome variable and DSST 1 (T4) as predictor | | | | | |
| --- | --- | --- | --- | --- | --- |
| Predictor | B | SE | p | OR | 95% CI |
| Intercept | -0.993 | 0.752 | 0.186 | 0.37 | 0.081-1.577 |
| DSST 1 | 0.020 | 0.014 | 0.170 | 1.02 | 0.992-1.050 |
| Nagelkerkes R^2^ =0.027 | | | | | |
| DSST - Digit Symbol Substitution Test | | | | | |

| **Table A15:** Bivariate logistic regression analyses with RTW (yes/no) (T4) as outcome variable and DSST 2 (T4) as predictor | | | | | |
| --- | --- | --- | --- | --- | --- |
| Predictor | B | SE | p | OR | 95% CI |
| Intercept | -1.049 | 0.575 | 0.068 | 0.350 | 0.108-1.051 |
| DSST 2 | 0.180 | 0.091 | 0.049 | 1.197 | 1.005-1.440 |
| Nagelkerkes R^2^ =0.056 | | | | | |
| DSST - Digit Symbol Substitution Test | | | | | |

| **Table A16:** Bivariate logistic regression analyses with RTW (yes/no) (T4) as outcome variable and SPSH (T4) as predictor | | | | | |
| --- | --- | --- | --- | --- | --- |
| Predictor | B | SE | p | OR | 95% CI |
| Intercept | -2.199 | 0.128 | <0.001 | 0.111 | 0.086-0.142 |
| SPSH | 0.195 | 0.012 | <0.001 | 1.215 | 1.187-1.244 |
| Nagelkerkes R^2^ =0.124 | | | | | |
| SPSH - subjective perceived status of physical and mental health | | | | | |

| **Table A17:** Bivariate logistic regression analyses with RTW (yes/no) (T4) as outcome variable and MVPA (T4) as predictor | | | | | |
| --- | --- | --- | --- | --- | --- |
| Predictor | B | SE | p | OR | 95% CI |
| Intercept | -0.868 | 0.493 | 0.078 | 0.420 | 0.152-1.066 |
| MVPA | 0.015 | 0.008 | 0.058 | 1.016 | 1.000-1.033 |
| Nagelkerkes R^2^ =0.067 | | | | | |
| MVPA - moderate to vigorous activity | | | | | |

| **Table A18:** Bivariate logistic regression analyses with RTW (yes/no) (T4) as outcome variable and PIA (T4) as predictor | | | | | |
| --- | --- | --- | --- | --- | --- |
| Predictor | B | SE | p | OR | 95% CI |
| Intercept | 0.241 | 1.616 | 0.881 | 1.273 | 0.052-32.191 |
| PIA | -0.019 | 0.113 | 0.868 | 0.981 | 0.783-1.227 |
| Nagelkerkes R^2^ =0.000 | | | | | |
| PIA - physical inactivity | | | | | |
