# Supplementary material for: Long-term course and factors influencing work ability and return to work in post-COVID patients 12 months after inpatient rehabilitation
Source: J Occup Med Toxicol. 2024 Nov 1;19:43. doi: 10.1186/s12995-024-00443-4 (PMC11529184; doi:10.1186/s12995-024-00443-4)
Supplement: Supplementary file 2 — Supplementary Material 2 [file 12995_2024_443_MOESM2_ESM.docx]

**Additional file 2**

**Self-generated questionnaire: The subjective perceived status of physical and mental health (SPSH)**

1. **Bitte bewerten Sie Ihre aktuelle körperliche Leistungsfähigkeit vor der Rehabilitation.**

| **Bitte bewerten Sie auf einer Skala von**  **0 (= sehr schlecht) bis**  **10 (= sehr gut)** | **0** | **1** | **2** | **3** | **4** | **5** | **6** | **7** | **8** | **9** | **10** |
| --- | --- | --- | --- | --- | --- | --- | --- | --- | --- | --- | --- |
| 1. Ihre aktuelle **all-gemeine körperliche Leistungsfähigkeit** | □ | □ | □ | □ | □ | □ | □ | □ | □ | □ | □ |
| 1. Ihre aktuelle **Atmungsfähigkeit** | □ | □ | □ | □ | □ | □ | □ | □ | □ | □ | □ |
| 1. Ihre aktuelle **Gehfähigkeit** | □ | □ | □ | □ | □ | □ | □ | □ | □ | □ | □ |
| 1. Ihre aktuelle **Ausdauerleistung** | □ | □ | □ | □ | □ | □ | □ | □ | □ | □ | □ |
| 1. Ihre aktuelle **Kraftfähigkeit in den Beinen** | □ | □ | □ | □ | □ | □ | □ | □ | □ | □ | □ |
| 1. Ihre aktuelle **Beweglichkeit** | □ | □ | □ | □ | □ | □ | □ | □ | □ | □ | □ |
| 1. Ihre aktuelle **Gleichgewichts-fähigkeit** | □ | □ | □ | □ | □ | □ | □ | □ | □ | □ | □ |
| 1. Ihre aktuelle **Kraftfähigkeit in den Händen** | □ | □ | □ | □ | □ | □ | □ | □ | □ | □ | □ |
| 1. Ihre aktuelle **Feinmotorik in den Händen** | □ | □ | □ | □ | □ | □ | □ | □ | □ | □ | □ |
| **Bitte bewerten Sie auf einer Skala von**  **0 (= nicht vorhanden) bis**  **10 (= stark ausgeprägt)** | **0** | **1** | **2** | **3** | **4** | **5** | **6** | **7** | **8** | **9** | **10** |
| 1. Ihre aktuelle **Post-COVID-Symptomatik** (z.B. Müdigkeit, Kopf-schmerzen, Husten, etc.) | □ | □ | □ | □ | □ | □ | □ | □ | □ | □ | □ |

1. **Bitte bewerten Sie Ihre aktuelle psychische Gesundheit in den letzten 2 Wochen.**

| **Bitte bewerten Sie auf einer Skala von**  **0 (= sehr schlecht) bis**  **10 (= sehr gut)** | **0** | **1** | **2** | **3** | **4** | **5** | **6** | **7** | **8** | **9** | **10** |
| --- | --- | --- | --- | --- | --- | --- | --- | --- | --- | --- | --- |
| 1. Ihre aktuelle **all-gemeine psychische Gesundheit** | □ | □ | □ | □ | □ | □ | □ | □ | □ | □ | □ |
| 1. Ihre aktuelle **Konzentrations-fähigkeit** | □ | □ | □ | □ | □ | □ | □ | □ | □ | □ | □ |
| 1. Ihre aktuelle **Emotionsregulation** | □ | □ | □ | □ | □ | □ | □ | □ | □ | □ | □ |
| 1. Ihre aktuelle **Schlafqualität** | □ | □ | □ | □ | □ | □ | □ | □ | □ | □ | □ |
| 1. Ihre aktuelle **Stimmung** | □ | □ | □ | □ | □ | □ | □ | □ | □ | □ | □ |
| 1. Ihre aktuelle **Motivation, etwas zu tun** | □ | □ | □ | □ | □ | □ | □ | □ | □ | □ | □ |
| 1. Ihre aktuelle **Selbstfürsorge** (wertschätzende Haltung und Handlungen sich selbst gegenüber / für sich selbst) | □ | □ | □ | □ | □ | □ | □ | □ | □ | □ | □ |
| 1. Ihren aktuellen **Appetit** | □ | □ | □ | □ | □ | □ | □ | □ | □ | □ | □ |
| **Bitte bewerten Sie auf einer Skala von**  **0 (= nicht vorhanden) bis**  **10 (= stark ausgeprägt)** | **0** | **1** | **2** | **3** | **4** | **5** | **6** | **7** | **8** | **9** | **10** |
| 1. Ihre aktuelle **Ängstlichkeit** | □ | □ | □ | □ | □ | □ | □ | □ | □ | □ | □ |
| 1. Ihre aktuelle **Müdigkeit** **/ Erschöpfung** | □ | □ | □ | □ | □ | □ | □ | □ | □ | □ | □ |
| 1. Ihr aktuelles **Gedankenkreisen** | □ | □ | □ | □ | □ | □ | □ | □ | □ | □ | □ |
